# Identification and Characterization of Fusolisin, the *Fusobacterium nucleatum* Autotransporter Serine Protease

**DOI:** 10.1371/journal.pone.0111329

**Published:** 2014-10-30

**Authors:** Lior Doron, Shunit Coppenhagen-Glazer, Yara Ibrahim, Amir Eini, Ronit Naor, Graciela Rosen, Gilad Bachrach

**Affiliations:** Institute of Dental Sciences, Hebrew University-Hadassah School of Dental Medicine, Jerusalem, Israel; Infectious Disease Research Institute, United States of America

## Abstract

*Fusobacterium nucleatum* is an oral anaerobe associated with periodontal disease, adverse pregnancy outcomes and colorectal carcinoma. A serine endopeptidase of 61–65 kDa capable of damaging host tissue and of inactivating immune effectors was detected previously in *F. nucleatum*. Here we describe the identification of this serine protease, named fusolisin, in three oral *F. nucleatum* sub-species. Gel zymogram revealed fusobacterial proteolytic activity with molecular masses ranging from 55–101 kDa. All of the detected proteases were inhibited by the serine protease inhibitor PMSF. analysis revealed that all of the detected proteases are encoded by genes encoding an open reading frame (ORF) with a calculated mass of approximately 115 kDa. Bioinformatics analysis of the identified ORFs demonstrated that they consist of three domains characteristic of autotransporters of the type Va secretion system. Our results suggest that the *F. nucleatum* fusolisins are derived from a precursor of approximately 115 kDa. After crossing the cytoplasmic membrane and cleavage of the leader sequence, the C-terminal autotransporter domain of the remaining 96–113 kDa protein is embedded in the outer membrane and delivers the N-terminal S8 serine protease passenger domain to the outer cell surface. In most strains the N-terminal catalytic 55–65 kDa domain self cleaves and liberates itself from the autotransporter domain after its transfer across the outer cell membrane. In *F. nucleatum* ATCC 25586 this autocatalytic activity is less efficient resulting in a full length membrane-anchored serine protease. The mature serine protease was found to cleave after Thr, Gly, Ala and Leu residues at the P1 position. Growth of *F. nucleatum* in complex medium was inhibited when serine protease inhibitors were used. Additional experiments are needed to determine whether fusolisin might be used as a target for controlling fusobacterial infections.

## Introduction

*Fusobacterium nucleatum* is a ubiquitous oral anaerobic rod classified into five subspecies *nucleatum*, *polymorphum*, *vincentii*, *animalis*, and *fusiforme* [1]. Development of periodontal disease has been correlated with a sharp increase in the numbers of *F. nucleatum* [2], [3]. *F. nucleatum* has a remarkable ability to attach to a range of early and late colonizing oral species [4], [5], [6], [7], [8], [9] in a process termed coaggregation or coadherence, and has therefore been suggested as a bridging organism that contributes to the structural formation of the multi-species dental biofilm [6], [10].

Virulence mechanisms of *F. nucleatum* include adhesion to and invasion of host cells [11] and induction of proinflammatory cytokines [12], [13]. *F. nucleatum* is also the periopathogen most commonly found in systemic infections [2]. It is strongly implicated in preterm deliveries [14], [15], and was also found to be dominant in the microenvironment of colorectal carcinoma [16], [17] and to promote its acceleration [18], [19].

Bacterial pathogens have developed strategies to enable their survival and growth within their specific hosts. Surface and secreted proteases are common virulence factors employed by microorganisms for colonization of new sites within the host, acquisition of growth nutrients and evasion of the host defenses [20]. Serine proteases are the most abundant and functionally diverse group of proteolytic enzymes in eukaryotic and prokaryotic organisms [21]. A family of extracellular serine proteases secreted through the Type V autotransporter secretion pathway, has been described in pathogenic Gram-negative species of *Neisseria*, *Shigella*, *Escherichia coli*, *Citrobacter rodentium*, *Salmonella* and *Edwarsiella* species [22]. These bacterial serine proteases hydrolyze host intracellular and extracellular protein substrates leading to cytoskeleton destruction [23], [24], induction of autophagy [25], [26] or impaired immunity [27].

Oral bacteria found in the subgingival plaque are predominantly anaerobic and rely on the utilization of peptides and amino acids for energy [28], [29]. The proteases of these oral microorganisms are implicated in the degradation of host periodontal tissues while supplying the bacteria’s nutritional requirements [30], [31].

Amino acids and peptides are the preferred substrates for *F. nucleatum’s* growth [32], [33], [34] and growth of fusobacteria depends on the availability of free glutamate, histidine, serine and lysine [35]. Under natural conditions, the above amino acids are not found in free form but are incorporated in proteins that have to be degraded for the desired amino acids to become accessible.

Previous studies reported a fusobacterial serine protease activity associated with a molecular mass of 65 kDa [36], [37], [38], [39]. This protease was shown to be capable of degrading components of periodontal tissues, and to inactivate host defense effectors [39]. The aim of this study was to identify and characterize the *F. nucleatum* 65 kDa serine protease which we named fusolisin.

## Materials and Methods

### Bacteria and growth conditions

*F*. *nucleatum* ATCC 10953 (subsp. *polymorphum*), ATCC 25586 (subsp. *nucleatum*), ATCC 49256 (subsp. *vincentii*) and FDC 364 (16S rDNA closest homology to *F. nucleatum* JCM 6328 subsp. *nucleatum,* see below) and *Porphyromonas gingivalis* PK 1924 were a gift from Dr. P. E. Kolenbrander (NIH, Bethesda, MD). *F. nucleatum* ATCC 23726 (subsp. *nucleatum*) was a kind gift from Prof. S. K. Haake (UCLA, Los Angeles, CA). Strain 12230 (subsp. *polymorphum*) was a kind gift from Prof. Y. Han (Case Western Reserve University, Cleveland, OH).

The bacteria were grown under anaerobic conditions (N_2_:CO_2_:H_2_, 85∶5∶10) in a Bactron II anaerobic chamber (Sheldon Manufacturing Inc., Cornelius, OR) at 37°C in Wilkins Chalgren anaerobic broth (Fluka, Spain). Bacterial purity was determined by phase contrast microscopy and Gram staining.

*Escherichia coli* strain XL1 (Agilent Technologies, CA) used for plasmid construction and *E.coli* ATCC 25922 were grown in Luria-Bertani (LB) medium or on LB agar plates supplemented with chloramphenicol (35 µg/ml; Sigma-Aldrich, Germany) at 37°C under aerobic conditions.

### Culture supernatant and outer membrane vesicle preparation

Four-day-old *F. nucleatum* cultures were harvested by centrifugation at 10,000×g for 20 min at 4°C. Culture supernatants were collected and filtered through a 0.2 µm filter (Whatman Schleicher & Schuell, Germany). Supernatants were either concentrated×10 using a Centricon microconcentrator (50,000-molecular-weight cutoff; Amicon) or used for outer membrane vesicle preparation.

For vesicle preparation cell-free supernatants were centrifuged at 100,000×g for 2 hrs. The supernatant was discarded and the pellet containing the vesicles was washed twice with TBS by centrifugation at 100,000×g. The pellet was stored at −20°C until further use.

### Gel Electrophoresis

For zymogram analysis, samples were dissolved at room temperature in sample buffer (192 mM Tris-HCl [pH 6.8], 30% glycerol, 9% SDS) without β-mercaptoethanol and subjected to SDS-PAGE using 7.5% gels containing 240 µg/ml human fibrinogen (Sigma-Aldrich, Germany). Following electrophoresis, the gels were incubated for 30 min at room temperature in Tris-buffered saline (TBS, 0.05 M Tris-HCl [pH 7.8], 0.1 M NaCl), containing 2% Triton X-100 and then washed three times with TBS. Gels were incubated overnight at 37°C. Proteolytic activity was visualized as a clear band against a blue background after staining with Coomassie brilliant blue R-250 as described before [39].

For denaturing SDS-PAGE, samples were dissolved, boiled at 100°C for 5 min in sample buffer containing 2% β-mercaptoethanol and the gels were stained with Coomassie brilliant blue. Molecular masses of protein bands were calculated by linear regression analysis of molecular mass standards.

### Mass spectrometry (MS) identification and database searching

Bands were excised from denaturizing gels and subjected to Qtof2 (Micromass, Manchester, UK) equipped with a nanospray capillary [40], analyzed by electrospray ionization tandem mass spectrometry (ESI-MS/MS) and peptides were identified as described before [40], [41].

### DNA isolation

Chromosomal DNA was isolated from *F. nucleatum* ATCC 25586 using the mini GenElute Bacterial Genomic DNA kit (Sigma-Aldrich, Germany) according to the manufacturer’s instructions. Plasmid DNA was isolated using the Qiagen spin miniprep kit (Qiagen, Germany).

### Expression of Fsp25586 in *F. nucleatum* ATCC 23726

The DNA fragment containing *fsp25586* and 556 bp of its up-stream region was amplified using the F-25586-SP90 (5′-CCgagctcGGAGCTTGATTTACATCCAAG-3′) and R- 25586-SP90 (5′-CCgagctcACTAGTGTTAGTGACGCAA-3′) primers that include a *SacI* restriction site (small case letters). The 3.9 kb PCR product was restricted with *SacI* (New England Biolabs Inc. USA), and inserted into the *SacI* site of the pHS30 *E. coli-F. nucleatum* shuttle vector [42], [43] to generate pHSPROT. Plasmid electroporation into *F. nucleatum* ATCC 23726 was performed as described previously [43]. Clones were selected on Columbia agar plates supplemented with 5% sheep blood (Hylabs, Israel) and 5 µg/ml thiamphenicol (Sigma-Alderich, Germany).

### Sequencing of FN1426

DNA was isolated from *F. nucleatum* ATCC 25586 as described above. The following primers were used to amplify and sequence the FN1426 gene:

F-25586-SP90– CCGAGCTCGGAGCTTGATTTACATCCAAG
R-25586-SP90– CCGAGCTCACTAGTGTTAGTGACGCAA
F-IP-25586-SP90– AAGAGCTCGTAACCCTGTTGAGATTACTG
F-2Sq-FnPro – CTGTTGCTGATGTAAAGCCCAT
R-4Sq-FnPro – CCAACTGTAGCTAATCCTTTGG
F-MS-Sq-FnPro – GGTGATGTTTTTACTCTTCTCC
R-MS-Sq-FnPro – CGGAATTAGATGCTAGTCTTGC
R-PE-Sq-FnPro – GCCCAGTATTTGGAGTATATGG

### Sequencing of the 16S rDNA of *F. nucleatum* FDC364

The 16S rRNA gene of *F. nucleatum* FDC 364 was amplified by PCR using universal primers 4F (CCA GAG TTT GAT YMT GGC) and 1541R (GAA GGA GGT GWT CCA DCC). The resulting product was sequenced (gene bank accession number KM023647) and blasted against the National Center for Biotechnology Information (NCBI) database. Closest alignment was found with the partial sequence of 16S ribosomal RNA gene of *F. nucleatum* JCM 6328 subsp. *nucleatum* GI:307219163.

### Effect of serine protease inhibitors on growth of *F. nucleatum* and *E. coli*

Overnight cultures of *F. nucleatum* or *E. coli* ATCC 25922 were diluted to an optical density at 600 nm of 0.02 in the appropriate growth medium. The irreversible serine protease inhibitors Phenylmethanesulfonyl fluoride ((PMSF, Sigma-Aldrich, Germany) and 4-(2-Aminoethyl)benzenesulfonyl fluoride hydrochloride (AEBSF, Sigma-Aldrich, Germany) were prepared to a stock solution of 100 mM in anhydrous ethanol and DDW respectively.

When added, PMSF and AEBSF were used at a final concentration of 1 mM and 2 mM respectively. *P. gingivalis* supernatants were prepared by centrifugation of four day cultures at 10,000×g for 10 min at 4°C., collection of the supernatants and filtration through a 0.2 µm filter (Whatman Schleicher & Schuell, Germany). When added, *P. gingivalis* supernatants were diluted 1:10 in the tested reaction. The final reaction contained 180 µl of diluted bacteria in a total volume of 220 µl. Bacterial growth (anaerobic for *F. nucleatum* and aerobic for *E. coli*) was monitored using microplate real-time kinetic measurements as described by us in detail previously [44]. Results represent mean and standard deviation of triplicate of an independent representative experiment repeated three times.

### Identification of the fusolisin restriction site

Fusolisin was purified from extracellular vesicles by preparative SDS-PAGE followed by electroelution as described before [39]. Briefly, four-day-old *F*. *nucleatum* cultures were sedimented by centrifugation at 9000×*g* for 20 min. The supernatant was collected and filtered through a 0.2 µm filter. The filtrate was aliquoted and outer membrane vesicles were sedimented by centrifugation at 100,000×g for 2 h. The supernatant was discarded, and the precipitate containing the extracellular vesicles was washed twice with 50 mM Tris-HCl (pH 7.8) by centrifugation as described above. For fusolisin purification, the vesicles were subjected to electroelution after separation by SDS-PAGE as follows: the extracellular vesicles were dissolved in sample buffer (without β-mercaptoethanol, see above), centrifuged for 2 min at 10,000×*g* and submitted to SDS-PAGE (7.5% acrylamide). The protease was electroeluted from the gel using a Bio Trap 1000 electroeluter (Schleicher and Schuell, Germany) with Tris-glycine buffer (25–192 mM) without SDS for 2 h at 200 volts followed by 10 h at 100 volts. The fusolisin enzyme was then stored at –20°C.

Identification of the fusolisin substrate specificity was determined by hydrolysis of fibrinogen and identification of the resulting peptides by mass spectrometry. The purified enzyme (0.25 µg) was incubated with 2.5 µg of fibrinogen in 40 µl TBS pH 8.0 at 37°C for 16 h. A similar reaction mixture with heat inactivated protease (3 min at 100°C) served as control.

The reaction mixture of fusolisin-mediated hydrolysis of fibrinogen was submitted to peptide mapping after N-terminus labeling by reductive dimethylation as follows: the protein sample in 8 M Urea and 50 mM Hepes (pH 8) was modified with 20 mM formaldehyde in the presence of 100 mM NaCBH_3_ (60°C for 15 min). Neutralization was performed with 500 mM ammonium bicarbonate (final concentration). The protein sample was reduced with 2.8 mM DTT (60°C for 30 min), modified with 9.4 mM iodoacetamide in 100 mM ammonium bicarbonate (room temperature for 30 min in the dark) diluted 4 fold and digested with modified trypsin (Promega) overnight at 37°C in a 1∶50 enzyme-to-substrate ratio.

The resulting peptides were desalted on a stage tip (C18) and resolved by reverse-phase chromatography on 0.075×200-mm fused silica capillaries (J&W) packed with Reprosil reversed phase material (Dr Maisch GmbH, Germany). The peptides were eluted with linear 60 minutes gradients of 5 to 45% and 15 minutes at 95% acetonitrile with 0.1% formic acid in water at flow rates of 0.25 µl/min. On line mass spectrometry was performed by an ion-trap mass spectrometer (OrbitrapXL, Thermo) in a positive mode using repetitively full MS scan followed by collision induced dissociation (CID) of the 7 most dominant ions selected from the first MS scan.

The mass spectrometry data was analyzed using the Thermo Protein Discoverer 1.3 using the Sequest search engine vs a specific sequence or a general database (Uniprot).

The cleavage site of fusolisin was further characterized by hydrolysis of the FRETS-25 Thr fluorescence-quenching substrate library D-A2pr(Nma)- Gly- [Phe/Ala/Val/Glu/Arg] - [Pro/Tyr/Lys/Ile/Asp]- **Thr**- Ala- Phe- Pro-Lys(Dnp)- D-Arg- D-Arg TRIFLUOROACETATE (PeptaNova GmbH, Germany). The reaction mixture contained 0.1 mM FRETS-25 Thr and 1.2 µg of purified fusolisin in 100 µl TBS pH 8.0 at 37°C. A reaction mixture with heat-inactivated protease served as control. Cleavage was monitored (λex = 340 nm and λem = 440 nm) every 20 min using a GENios Microplate reader (TECAN, Austria). Results represent mean and standard deviation of three independent experiments.

Cleavage of FRETS-25 Thr was analyzed as described above but without the N-terminus labeling.

Fusolisin’s restriction specificity was verified using the FRET substrate CPQ2-Gly-Phe-Ile-Thr-Ala-Phe-Pro-Lys-(5FAM)-Arg-Arg-NH2 that was custom synthesized by CPC scientific (Sunnyvale, CA, USA). The peptide was dissolved in DMSO to a concentration of 1 mM and further diluted with TBS to the desired concentration. The reaction was performed and monitored as described above with the λex = 485 nm and the λem = 535 nm.

### Bioinformatics analysis

Public databases were searched for similar sequences with the BLASTN, BLASTP, and BLASTP/PSI algorithms using default parameters. The features of the predicted proteins were examined by the Pfam programs (http://www.sanger.ac.uk/Software/Pfam/search.shtml). The *ExPASy* server was used to predict the proteins' molecular weights [45]. Multiple alignment was performed using CLUSTAL W [46], [47]. Structure prediction was generated using the Protein Homology/analogY Recognition Engine (Phyre) [48]. Rare Codon Caltor (http://www.doe-mbi.ucla.edu/~sumchan/caltor.html) was used before cloning in *E. coli.*

## Results

### Identification of fusolisin

**Figure 1:**
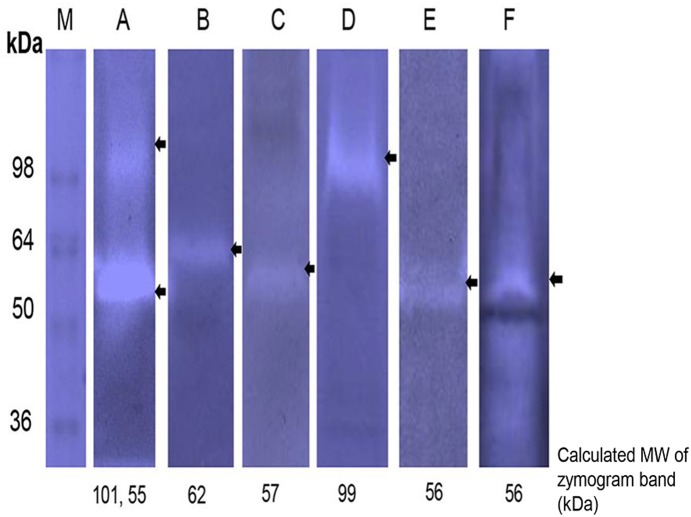
Protease profiles of *F. nucleatum* growth medium supernatants on fibrinogen containing zymograms. M, Molecular weight markers. A, *F. nucleatum* ATCC 49256. B, *F. nucleatum* FDC 364. C, *F. nucleatum* ATCC 10953. D, *F. nucleatum* ATCC 25586. E, *F. nucleatum* ATCC 23726. F, *F. nucleatum* 12230. Arrows indicate proteolytic bands. Presented data are of representative zymograms.

**Figure 2:**
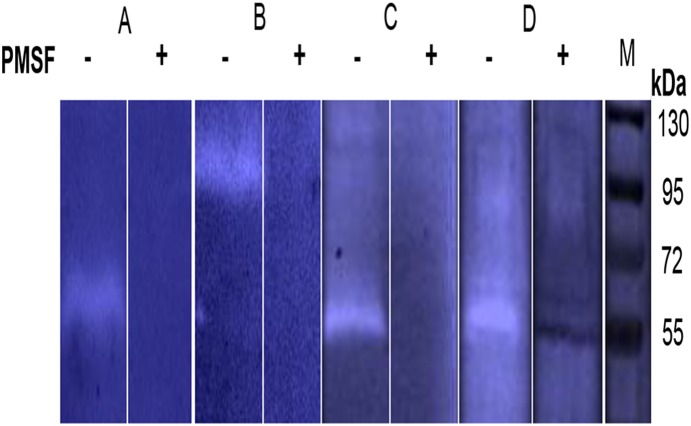
PMSF inhibits the proteolytic activity of *F. nucleatum*. A, *F. nucleatum* FDC 364. B, *F. nucleatum* ATCC 25586. C, *F. nucleatum* 12230. D, *F. nucleatum* ATCC 23726. M, Molecular weight markers. Presented data are of representative zymograms.

Gel zymograms using human fibrinogen as a substrate, revealed proteolytic activity in the growth media supernatant (Fig. 1) and in outer membrane vesicles prepared from all of the tested *F. nucleatum* strains that represent three *F. nucleatum* subspecies: *nucleatum*, *polymorphum* and *vincentii* (Table 1). The molecular weight of the detected proteases varied from 55 to 101 kDa as estimated by gel migration (Fig. 1, Table 1). All the detected proteases were inhibited by the serine protease inhibitor PMSF (presented for *F. nucleatum* strains FDC 364, ATCC 25586, 12230 and ATCC 23726 in Fig. 2).

**Table 1:** Estimated molecular mass of *F. nucleatum* fusolisin detected in outer membrane vesicles or in growth medium.

F. nucleatum strain	Name (GeneBank)	Estimatedmolecular mass ofproteolytic bands
**ATCC 49256**(subsp. vincentii)	**Fsp49256** **(FNV0835)**	5**5,** 101
**FDC 364**(subsp. nucleatum)	**Fsp364**	**62, 96** a
**ATCC 10953**(subsp. polymorphum)	**Fsp10953** **(FNP_2077)**	**5**7**,** 9**6** a
**ATCC 25586**(subsp. nucleatum)	**Fsp25586** **(**KJ634469, **suggested** **annotation for FN1426)**	9**9**
**ATCC 23726**(subsp. nucleatum)	**Fsp23726** **(**03970469****	**56, 96** a
**12230**(subsp. polymorphum)	**Fsp12230**	**56**

a Activity detected only in samples prepared from outer membrane vesicles.

**Figure 3:**
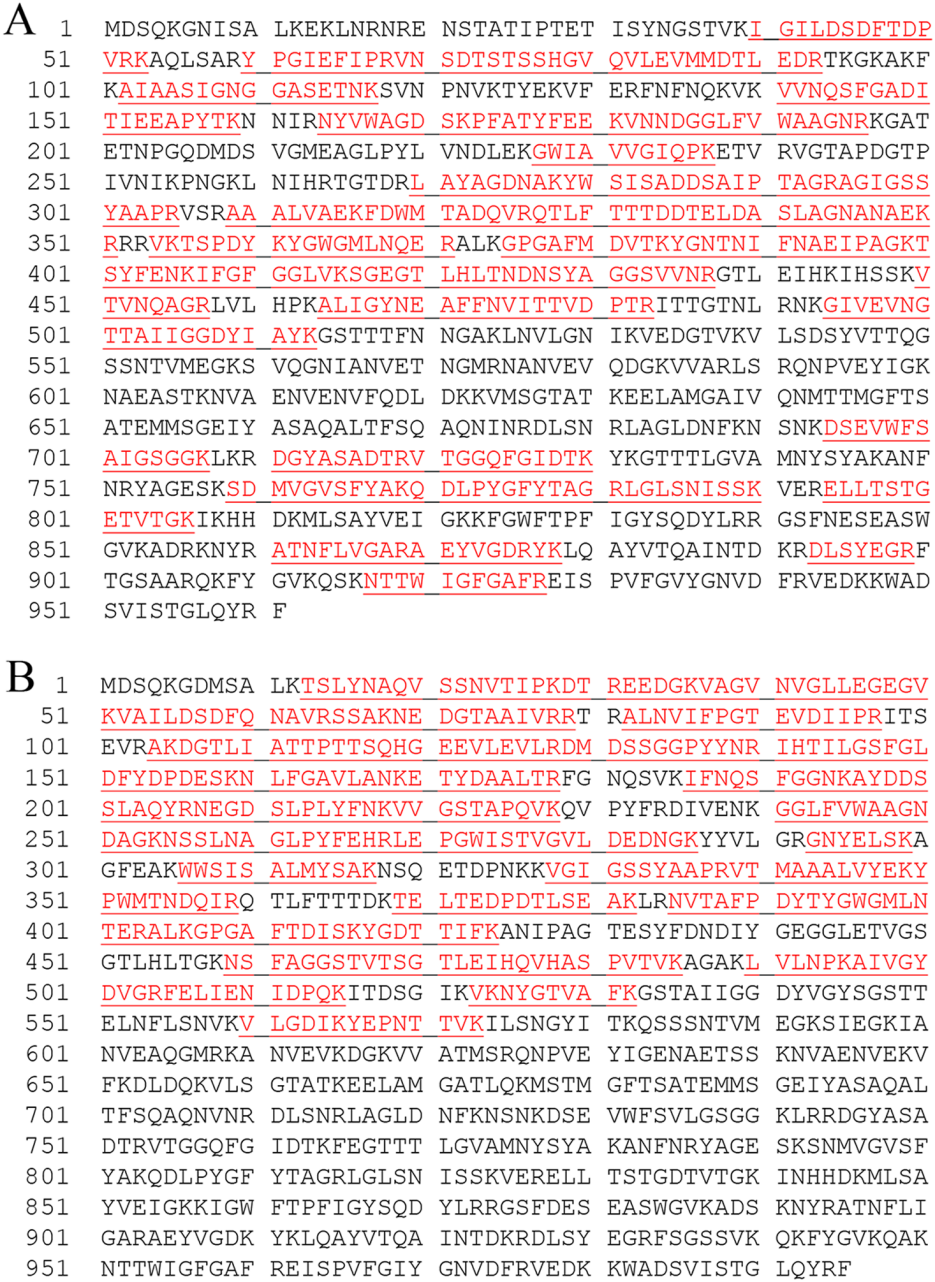
Identification of the fusobacterial serine protease. Amino acid sequences of the putative serine protease open reading frames FN1426 (Fsp25586) (A), and FNV0835 (Fsp49256) (B). Red highlight indicates sequences identified by mass spectrometry of the 99 kDa serine protease of *F. nucleatum* ATCC 25586 (A), and of the 55 kDa serine protease of *F. nucleatum* ATCC 49256 (B).

Gel-purified proteases of outer membrane vesicles prepared from the genome-sequenced *F. nucleatum* strains ATCC 25586 and ATCC 49256 were identified using mass spectrometry (MS). Tryptic fragments of the 99 kDa proteolytic protein of *F. nucleatum* ATCC 25586 matched those of the entire putative 115 kDa serine proteases designated FN1426 [Genbank Index number (GI):19704758]. Tryptic peptides of both the 55 kDa and 101 kDa serine endopeptidases partially purified from *F. nucleatum* ATCC 49256 were found to match those of the putative 108 kDa serine protease designated FNV0835 (GI:34763535). However, while the peptide sequences generated from the 99 kDa proteolytic protein extracted from *F. nucleatum* ATCC 25586 corresponded to the entire FN1426 protein sequence (Fig. 3A), those generated from the 55 kDa proteolytic band of *F. nucleatum* ATCC 49256 matched only the N-terminal domain of FNV0835 (Fig. 3B) suggesting that the 55 kDa protease of *F. nucleatum* ATCC 49256 also originated from a larger precursor. The 62 kDa protease of the non-sequenced strain FDC 364 [39] was found to be most homologous to the N-terminal domain of FN1426 of *F. nucleatum* ATCC 25586 (data not shown).

### Sequence analysis of fusolisin

Previous annotation of the FN1426 sequence of *F. nucleatum* ATCC 25586 [49], showed that the start codon proposed earlier by Kapatral and colleagues [50] has been misannotated. Upon detailed analysis, we found that the reading frame proposed by Kapatral and colleagues [50] was indeed truncated. However, we found that the reading frame extends even further than corrected by Desvaux and colleagues [49] which is missing 291 base pairs at the beginning of the gene. The FN1426 gene bank sequence begins with a TTG codon (an alternate start codon in bacteria), our proposed new open reading frame begins with ATG which is located 291 base pairs upstream. Furthermore, analysis of the sequence taken from the gene bank using the SignalP 4.0 server [51], found no signal peptide while analysis of our proposed sequence revealed a putative signal peptide cleavage site between amino acids 58 and 59. This observation was verified by PCR and sequencing of the proposed ORF and the 400 bases upstream to it. The corrected predicted 1,058 amino acid ORF, was named Fsp25586 (for Fusobacterial Serine Protease of *F. nucleatum* 25586) and was deposited in the gene bank accession number KJ634469. Henceforth, all of the following analysis will refer to the new sequence of Fsp25586. The orthologous sequences FNV0835 will be referred to as Fsp49256, FNP_2077 will be referred to as Fsp10953 and HMPREF0397_0469 will be referred to as Fsp23726.

**Figure 4:**
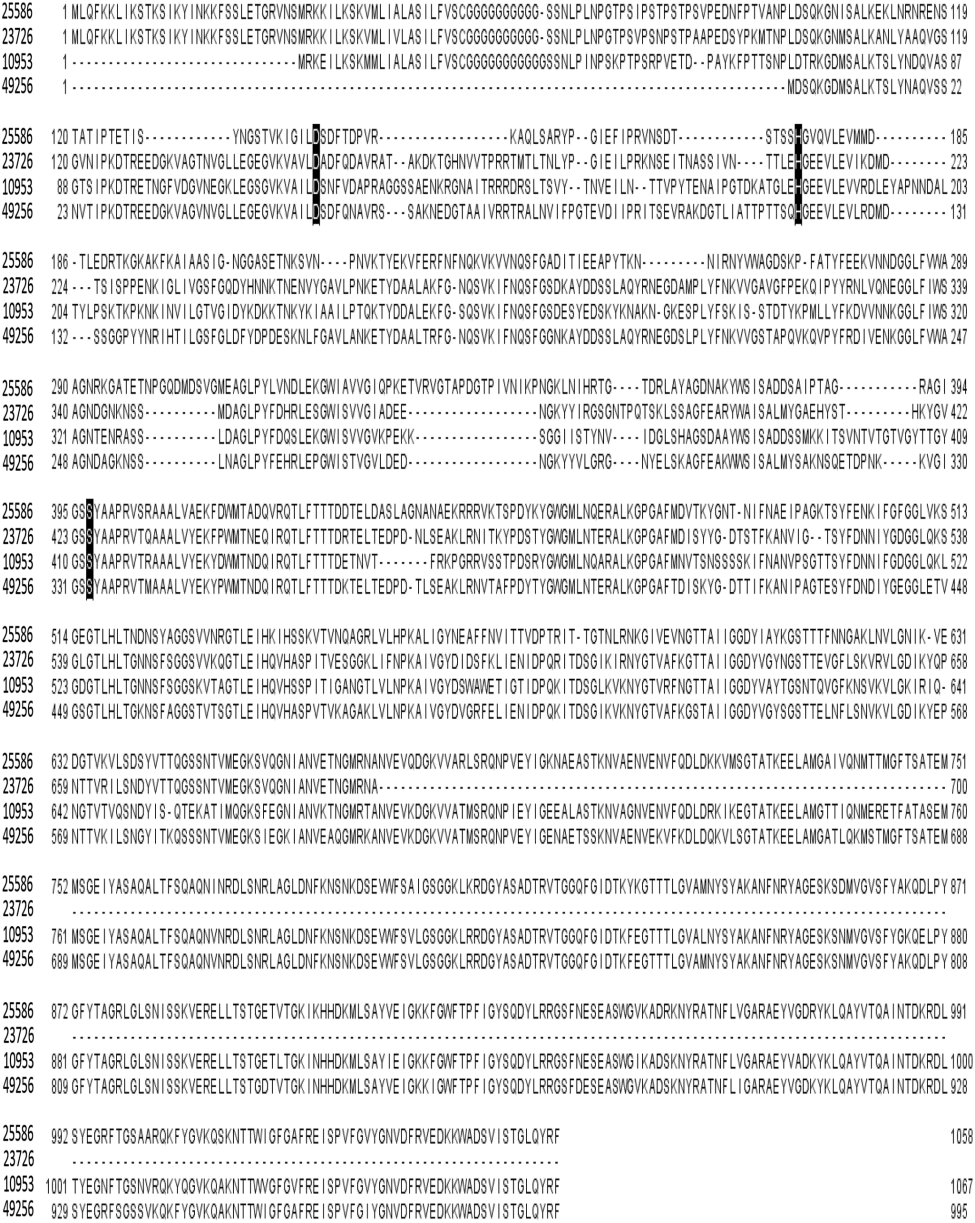
Sequence alignment of fusolisin. ClustalW alignment of Fsp25586, the available partial sequence of the homologues serine protease Fsp23726, Fsp10953 and Fsp49256. The predicted catalytic triad Asp, His and Ser are highlighted.

Amino acid sequence alignment (Fig. 4) of Fsp25586 revealed a high homology (71% similarity and 61% identity) with that of Fsp49256, 71% similarity and 60% identity with that of Fsp10953 and 63% similarity and 57% identity with the available partial sequence of the homologous serine protease Fsp23726. Previous annotation of the FN1426 (Fsp25586) and the FNV0835 (Fsp49256) open reading frames revealed a signal peptide and three other functional domains [49]. The N-terminal, peptidase domain [amino acids 131–471 in Fsp25586 and 1–406 in Fsp49256] were found to belong to the peptidase S8 domain family. The C-terminal domain (amino acids 788–1047 in Fsp25586 and 690–995 in Fsp49256) belong to the autotransporter superfamily. While the C-terminal autotransporter domain of Fsp25586 and Fsp49256 were highly conserved (93% identity and 98% similarity), a higher divergence was found between the catalytic domains of the proteases of the two species (37% identity, 47% similarity). As a member of the S8 family of subtilisins, the amino acid sequence analysis of fusolisin revealed that the arrangement of the active site catalytic triad is Asp-His-Ser [52], [53] that was identified using NCBI's conserved domain database (CDD) [54] in the amino acid sequences of Fsp49256, Fsp10953, Fsp23726 and Fsp25586, and can be seen in Fig. 4.

### Processing of fusobacterial serine protease

**Figure 5:**
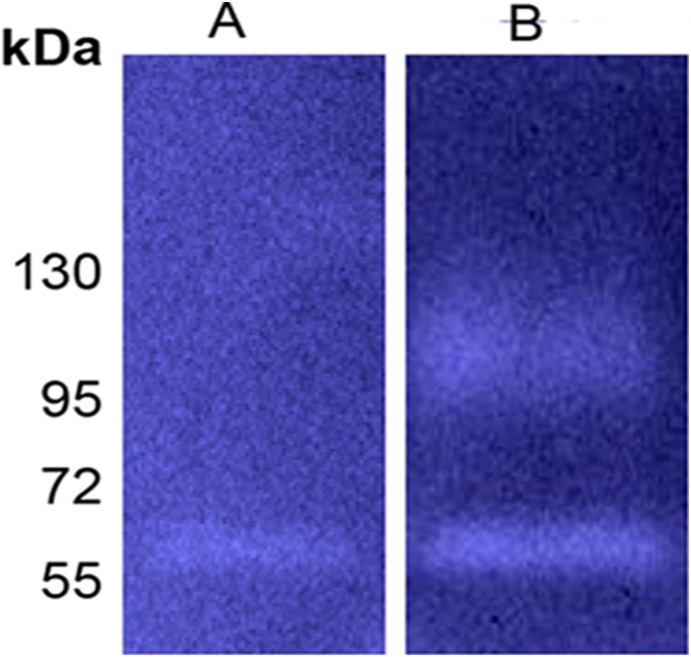
Self-restriction of Fsp25586 is not efficient. Zymogram analysis of cell culture supernatant prepared from *F. nucleatum* ATCC 23726 carrying the pHS30 vector (A), or the pHSPROT plasmid expressing Fsp25586 (B).

Autocatalytic processing is common in type Va secretion systems [55], [56], [57] and in subtilisins [58]. Although *F. nucleatum* ATCC 25586 remains refractory to plasmid transformation, others and us were previously successful with plasmid expression in *F. nucleatum* ATCC 23726 [43], [59]. As can be seen in figures 1E and 5A, the serine protease detected in the growth medium of *F. nucleatum* ATCC 23726 (Fsp23726) is approximately 56 kDa. Zymogram analysis of culture supernatants prepared from *F. nucleatum* ATCC 23726 expressing Fsp25586 of strain ATCC 25586 revealed the presence of the 99 kDa Fsp25586 protease in addition to the typical 56 kDa protease of ATCC 23726 (Fig. 5B). The fact that Fsp25586 was not cleaved when expressed in *F. nucleatum* ATCC 23726 suggests that the processing of Fsp25586 is not efficient compared to that in Fsp23726 (and the orthologs in the other tested *F. nucleatum* strains, Table 1). It is possible that Fsp25586 lacks the restriction site that is cleaved to release the catalytic domain from the autotransporter domain, or that this cleavage site is not exposed for cleavage.

### Fusolisin’s cleavage site

Previous work in our laboratory failed to determine the substrate specificity of the fusobacterial proteolytic activity using a large variety of synthetic chromogenic substrates [39]. We now determined the cleavage sites of fusolisin by MS analysis of the peptides resulting from hydrolysis of fibrinogen. Fusolisin cleaved fibrinogen preferentially at the C- terminal side of small residues (Thr, Gly, Ala and Ser), though Leu and Asp were also cleaved (Table 2). However, the major peak of fusolisin mediated fibrinogen hydrolysis resulted from cleavage of Thr at the P1 position.

**Table 2:** Substrate specificity of fusolisin.

A	
**Fibrinogen**	Peptide Intensity
GTAW**T**/A	4.5×10^9^
SGSSG/P	9.0×10^8^
TAWTA/D	4.0×10^8^
LGGWL/L	3.8×10^8^
NFNR**T**/W	3.8×10^8^
AWTAD/S	1.6×10^8^
PRNPS/S	2.6×10^7^
**B**	
**FRETS-25 Thr**	Peptide Intensity
T/AFPKRR	3.6×10^8^
GFIT/A	8.6×10^7^
GVIT/A	4×10^7^
GEIT/A	2.4×10^7^
GFPT/A	1.6×10^7^

A) Major peptides obtained by fusolisin hydrolysis of fibrinogen.

B) Major peptides obtained by fusolisin hydrolysis of FRETS-25 Thr.

D-A_2_pr(Nma)- Gly- [Phe/Ala/Val/Glu/Arg] - [Pro/Tyr/Lys/Ile/Asp]- **Thr**- Ala- Phe- Pro- Lys(Dnp)- D-Arg- D-Arg TRIFLUOROACETATE.

Mass spectrometry was performed as described in Materials and Methods. The peptide intensity based on the peak area was analyzed by LC-MS and ordered by decreasing abundance.

To verify that fusolisin preferentially cleaves Thr at the P1 position, hydrolysis of the FRETS-25 Thr substrate (PeptaNova) by fusolisin was examined. FRETS-25 Thr, is a protease substrate library that contains a highly fluorescent 2-(N-methylamino)benzoyl (Nma) group linked to the side chain of the amino-terminal D-2,3-diamino propionic acid (D-A2pr) residue, along with a 2,4-dinitrophenyl (Dnp) group that acts as a quencher, linked to the **ε**–amino group of a Lys residue. The fluorophore and quencher are connected by the peptide Gly- [Phe/Ala/Val/Glu/Arg] - [Pro/Tyr/Lys/Ile/Asp]- **Thr**- Ala- Phe- Pro. Mass spectrometry analysis of the Fsp23726-mediated FRETS-25 Thr hydrolysis products revealed that similar to fibrinogen cleavage, the major peaks obtained resulted from Thr in the P1 position (Table 2B). Preference for the presence of Ile in the P2 position was also observed (Table 2B).

**Figure 6:**
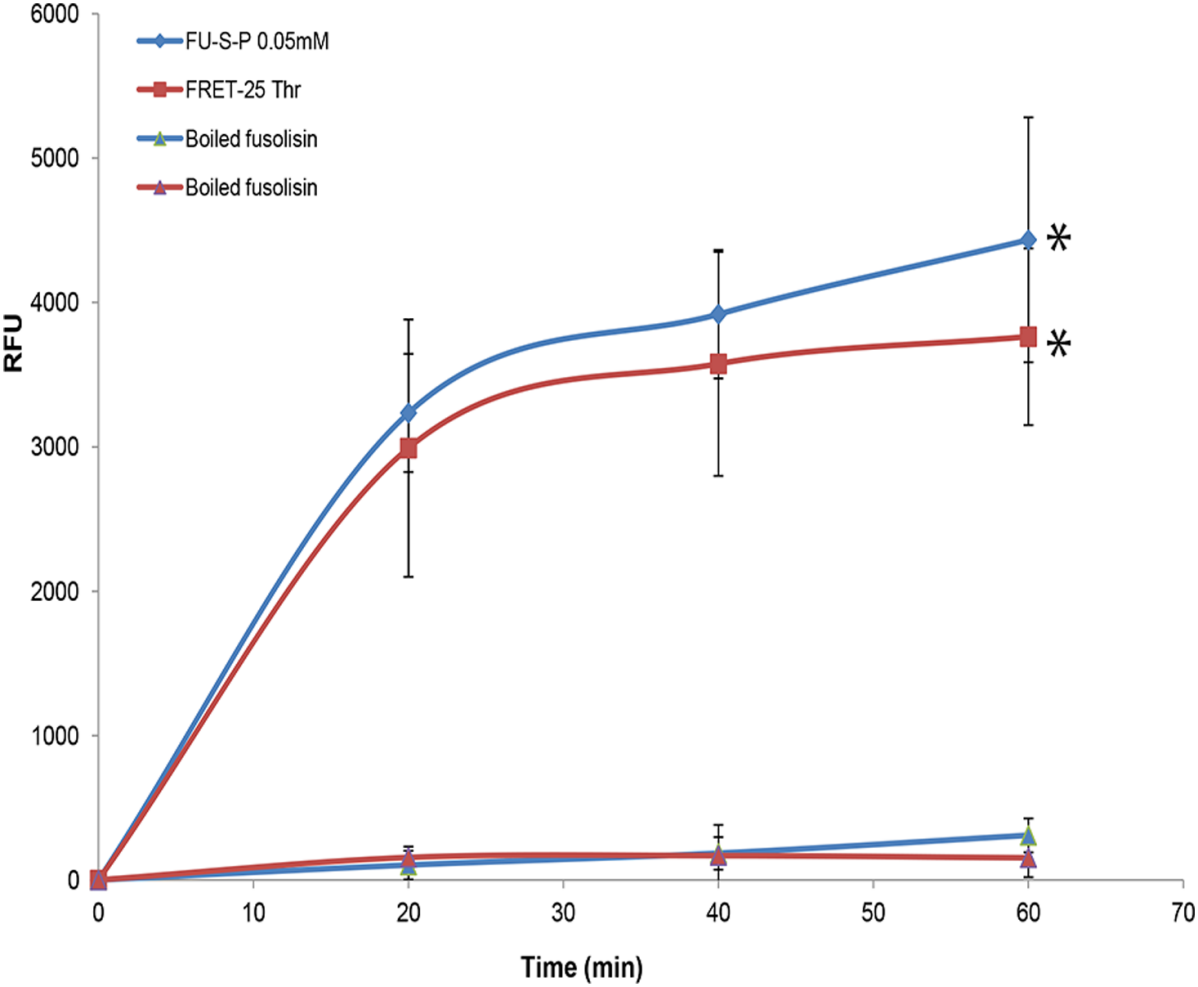
Time course hydrolysis of FRETS-25-Thr and Fu-S-P by fusolisin. Purified fusolisin (1.2 µg) was incubated with 0.05 mM of Fu-S-P or 0.1 mM (blue) of FRETS-25-Thr (red) in TBS pH 8.0. Relative Fluorescent Units (RFU) were determined as described in materials and methods. *P<0.05 compared to control with heat inactivated fusolisin, determined with Bonferroni test for multiple comparisons using the SPSS 15.0 software.

Based on the FRETS-25 Thr hydrolysis results, the following fusolisin sensitive FRET peptide, (Fu-S-P) was synthesized:

CPQ2-Gly-Phe-Ile-Thr-Ala-Phe-Pro-Lys-(5FAM)-Arg-Arg-NH2. Time course hydrolysis of FRETS-25 Thr and Fu-S-P by purified fusolisin is shown in Fig. 6.

**Figure 7:**
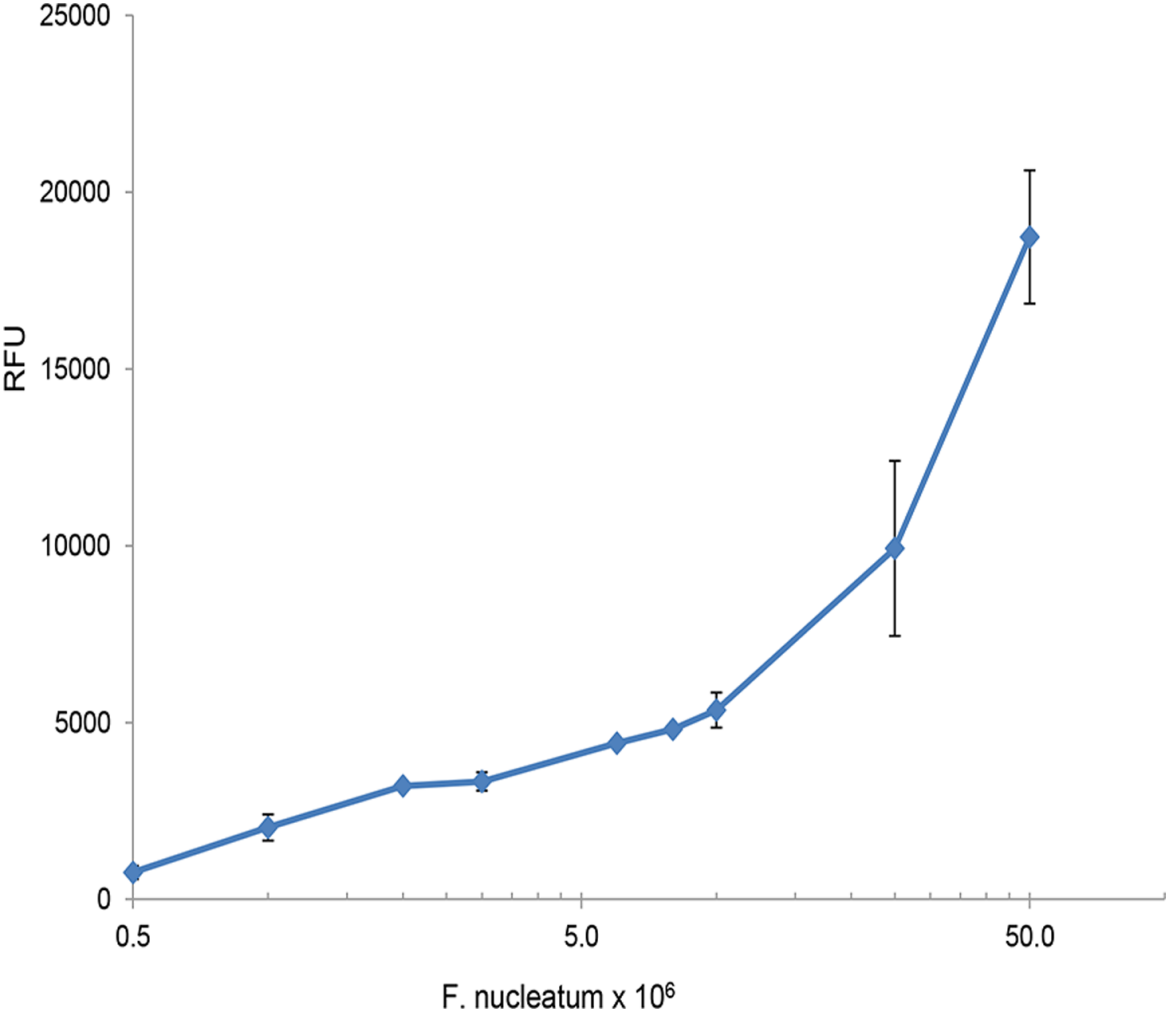
Fu-S-P activity correlates with the number of *F. nucleatum* cells. Fu-S-P (0.03 mM) was incubated for 2 hrs with increasing numbers of washed *F. nucleatum* cells. Relative Fluorescent Units (RFU) were determined as described in Materials and Methods. No activity was observed with boiled cells.

Fu-S-P was found to be a useful biomarker for detecting *F. nucleatum* ATCC 25586 (not shown) and ATCC 23726. As shown in Fig. 7, 5×10^5^–1×10^6^ fusobacterial cells were sufficient to produce measurable activity after 2 hrs.

### Fusolisin is essential for growth of *F. nucleatum* in a complex medium

**Figure 8:**
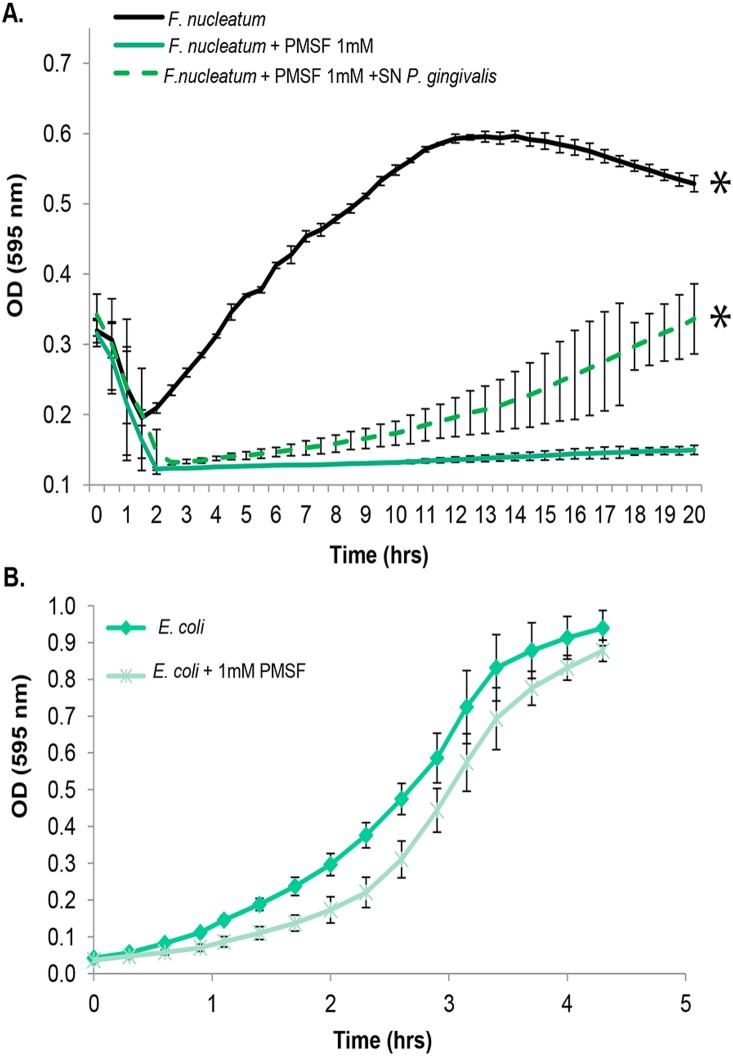
PMSF inhibits growth of *F. nucleatum* but not of *E. coli*. (A) Growth of *F. nucleatum* 12230 (black line) is inhibited by PMSF (solid green line), but this inhibition is relieved by *P. gingivalis* supernatant (SN Pg) containing PMSF-resistant cysteine proteases (broken green line). (B) Growth of *E. coli* is not affected by PMSF, ruling out PMSF toxicity. *P<0.05 compared to PMSF-treated bacteria, determined with Bonferroni test for multiple comparisons using the SPSS 15.0 software.

The genes coding for Fsp25586 and Fsp49256 were located in genomic loci involved in metabolic functions suggesting a nutritional role for fusolisin. Indeed, growth of *F. nucleatum* in a complex medium was inhibited by the AEBSF (not shown) and PMSF serine protease inhibitors (Fig. 8). Both inhibitors did not affect growth of *E. coli* used as control (Fig. 8), ruling out that growth attenuation of *F. nucleatum* by both serine-protease inhibitors resulted from non specific toxicity. *P. gingivalis* is a proteolytic anaerobic periodontal pathogen frequently isolated together with *F. nucleatum* [3]. The gingipain proteases produced by *P. gingivalis* are cysteine proteases which are not inhibited by PMSF. Addition of filter-sterilized gingipain-containing supernatant collected from a *P. gingivalis* culture [60], relieved the PMSF inhibitory effect on *F. nucleatum*’s growth (Fig. 8). Addition of the *P. gingivalis* supernatant did not affect the pH of the reaction mixture and did not reduce the inhibitory activity of PMSF as tested on trypsin under similar conditions (data not shown). These results suggest that *P. gingivalis* can enable fusolisin-independent growth of *F. nucleatum.*

## Discussion

**Figure 9:**
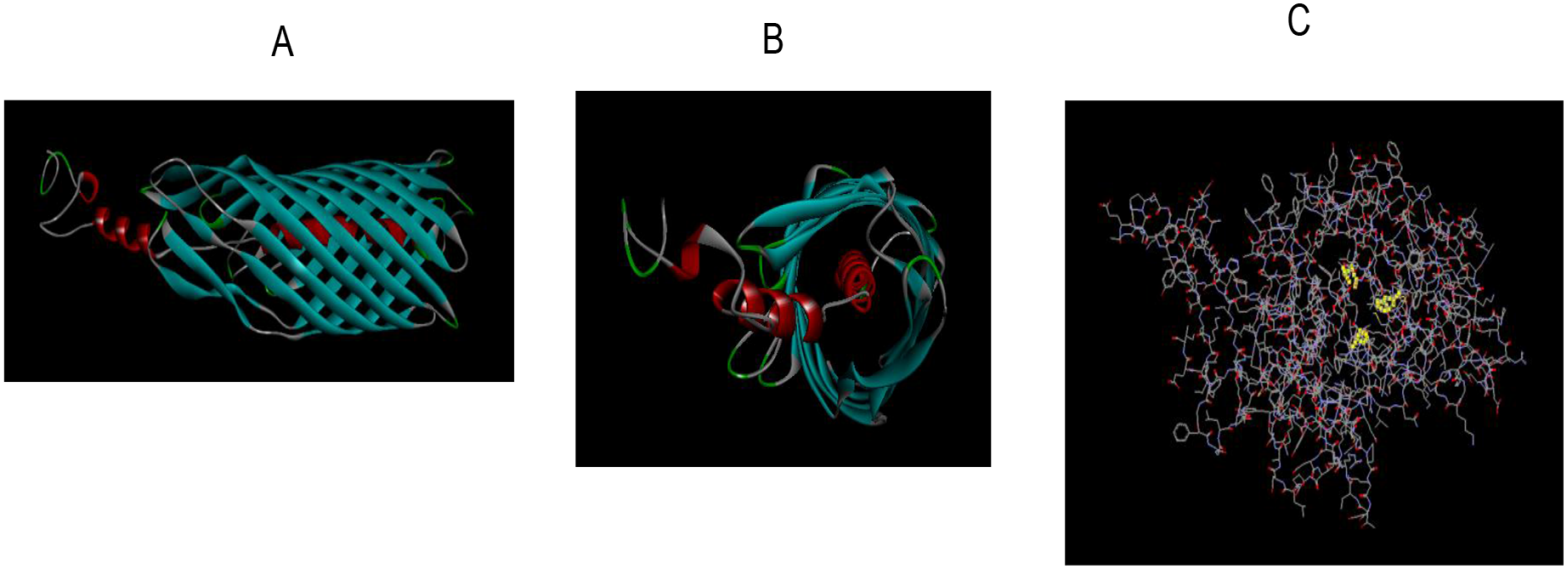
Fusolisin Fsp25586 analysis using the Protein Homology/analogY Recognition Engine (phyre). A) Side view of the autotransporter domain. B) Front view of the autotransporter domain. C) The catalytic domain with the characteristic serine protease catalytic triad Asp 141, His 175, and Ser 397 highlighted in yellow.

Obtaining energy by the fermentation of a small number of peptide-derived amino acids was shown to be essential for the growth of *F. nucleatum* [32], [34], [61]. To date, the only detected endopeptidase activity in *F. nucleatum* was that of an un-identified serine protease with a molecular weight of 61–65 kDa [36], [37], [38], [39]. In the present work this protease, now named fusolisin, has been identified and characterized at the genetic level. The theoretic isoelectric point of the 55 kDa derivative of Fsp49256 is 5. This calculated isoelectric point is in agreement with that (pH 5) determined for the 65 kDa diisopropylfluorophosphate-binding outer membrane protein of *F. nucleatum* Fev1 [37]. Mass spectrometry analysis and identification of the high (99 kDa) and the low (55 kDa) molecular weight *F. nucleatum* serine proteases partially purified from strains ATCC 25586 and ATCC 49256 demonstrated (Fig. 3) that both originate from a precursor with a calculated molecular weight of approximately 115 kDa. Sequence analysis suggested that this precursor contains a signal peptide, a serine protease domain, a linker, and an autotransporter domain. Structure prediction of the serine protease and of the autotransporter domains was generated using the Protein Homology/analogY Recognition Engine (Phyre) [48] and can be seen in Figure 9 and in Movies S1 and S2.

Our experimental results and *in* *silico* predictions suggest that the fusobacterial serine protease belongs to the autotransporter proteins superfamily of the type Va secretion pathway [55], [56], [57]. This conclusion is in agreement with a previous *in* *silico* analysis of the *F. nucleatum* genome [49].

Our bioinformatics results (Fig. 4) suggest that the 101–55 kDa fusolisin proteases (Fig. 1, Table 1) all derive from a precursor of approximately 115 kDa. This precursor crosses the cytoplasmic membrane presumably via the Sec (Secretion) pathway where the signal peptide is removed. The C-terminal domain of the remaining approximately 96–101 kDa poly-peptide then forms a β-barrel pore structure in the outer bacterial membrane (Fig. 9A, Movie S2). The N-terminal serine endopeptidase domain is then most likely threaded through the autotransporter and transported across the outer membrane to the cell’s exterior surface. An alternative model suggests that several autotransporter domains oligomerize and form a wide channel that enables the transfer of the catalytic passenger domain [56].

In the case of Fsp25586, the protease remains intact (99 kDa, Table 1, Fig. 1). In the case of Fsp49256 and its homologs in strains ATCC 10953, FDC 364 and ATCC 23726 the protease can remain intact and cell bound, or the catalytic domain can self cleave the peptide bridge connecting both domains and release itself to form the detected 55–62 kDa protease (Table 1, Fig. 1). Mass spectrometry analysis of the self cleavage product of Fsp49256 suggests that the restriction site is located after amino acid 572 (Fig. 3A). Furthermore, the amino acid sequence GYIT (amino acids 578 to 581 in Fig. 3B) is present in the peptide bridge connecting the catalytic domain to the autotransporter in Fsp49256 and absent in the peptide bridge in Fsp25586 (Fig. 3A). This sequence is similar to the fusolisin P4-P1 cleavage site GFIT in Fu-S-P, indicating that GYIT may be the self cleavage restriction site of Fsp49256.

The 55–62 kDa form of fusolisin was found in the growth media but also in outer membrane vesicles (Table 1). This indicates that the autotransporter domain is not essential for adherence of the catalytic domain to the bacteria’s outer membrane surface. Hydrophobic interactions between hydrophobic sub-domains (not shown) in the catalytic domain and the membrane, or non-covalent interactions between the catalytic and autotransporter domains can enable such association [62]. In strains ATCC 23726, ATCC 10953 and FDC 364 the full-length (∼96 kDa) protease was detected mainly in outer membrane vesicle preparations but not in growth media where the 56–62 kDa protease was found. This indicates that unlike the secreted mature 55–62 kDa protease, the full length protease in these strains is mostly membrane bound. The release of the mature serine protease that can act on extracellular targets [39] can enable its diffusion to distant locations and presumably increase its effectiveness [63]. Regulation of cleavage and detachment of the passenger from the autotransporter domain has been demonstrated in IcsA of *Shigella flexneri* [64], and the Hap autotransporter of *Haemophilus influenzae* [65].

The self cleavage mechanism that determines *F. nucleatum* serine protease secretion is yet unknown. Possible regulation of this secretion mechanism by the bacteria (in response to nutritional needs or proteinaceous host defense challenges) remains to be determined.

The fusolisin’s autotransporter domain was found to be much more conserved than the catalytic one (Fig. 4). This can result from strict structural-functional requirements. An additional possibility is that being extracellular, the catalytic domain is under much greater immunological stress than the non-exposed intramembrane autotransporter domain. This constant immunological selective pressure might accelerate alterations in the catalytic domain’s amino acid sequence.

Previous attempts to find the substrate specificity of fusolisin using a large number of chromogenic substrates have failed [39]. In the present study we synthesized a fusolisin substrate that allows the detection of low numbers of fusobacterial cells (Fig. 7). Such a substrate might be made useful for detection of fusobacteria associated with colorectal cancer [17], [18].

In order to survive in the host, fusobacteria need to overcome the host immune system and to acquire nutrients. Our previous results demonstrated that the *F. nucleatum* serine protease is capable of inactivating host immune mediators [39]. Our current study demonstrates the essential nutritional role of fusolisin. This was concluded from the fact that fusolisin inhibition restricted growth of *F. nucleatum* (Fig. 8A). In the oral plaque, *F. nucleatum* is always found in a multispecies environment that contains proteolytic members [3] that are likely to relieve the dependency on fusolisin for fusobacterial growth. Indeed, addition of filter-sterilized supernatant collected from a *P. gingivalis* culture that contains the gingipain cysteine proteases (which are not inhibited by PMSF) relieved the PMSF inhibitory effect on *F. nucleatum*’s growth (Fig. 8A).

*F. nucleatum* is frequently isolated or identified by molecular methods in the amniotic fluid of preterm births [15], [66], [67]. In some of these isolations *F. nucleatum* was found as a sole bacterium [68]. In this extraoral environment, and without the proteolytic oral bacterial neighbors, it is plausible that fusobacterial proliferation in the placenta is fusolisin dependent. It is therefore tempting to speculate that inhibition of fusolisin might affect the ability of *F. nucleatum* to translocate from the multispecies oral cavity and colonize the placenta.

## Supporting Information

Movie S1 **Structure prediction of serine protease catalytic domain of Fsp25586 (catalytic triad marked) generated using the Protein Homology/analogY Recognition Engine (Phyre).**
(MOV)

Movie S2 **Structure prediction of the autotransporter domain of Fsp25586 generated using the Protein Homology/analogY Recognition Engine (Phyre).**
(MOV)
